# Self-Blame Mediates the Link between Childhood Neglect Experiences and Internalizing Symptoms in Low-Risk Adolescents

**DOI:** 10.1007/s40653-020-00307-z

**Published:** 2020-04-29

**Authors:** Michal Tanzer, George Salaminios, Larisa Morosan, Chloe Campbell, Martin Debbané

**Affiliations:** 1 Psychoanalysis Unit, Research Department of Clinical, Educational and Health Psychology, University College London, 1-19 Torrington Place, London, WC1E 7HB UK; 2 Developmental Clinical Psychology Unit, Faculty of Psychology and Educational Sciences, University of Geneva, Geneva, Switzerland

**Keywords:** Neglect, Cognitive emotion regulation, Self-blame, Internalizing symptoms, Adolescence

## Abstract

Childhood neglect is the most common type of maltreatment, ranging from minor isolated incidents to consistent failures in emotional/physical caregiving. It has been associated with developmental impairments and considered a risk factor for the emergence of psychopathology, particularly internalizing disorders. This study aimed to explore individual differences in response to the continuum of severity of neglect in community adolescents, as well as the role of specific cognitive emotion regulation strategies (CERSs) as mediators between childhood neglect and current internalizing symptoms. Low-risk adolescents (12–19 years old; *M* age 15.88 years; *N* = 123; 64 Females) completed questionnaires assessing these experiences. We employed a regression model and a simple mediation analyses. Findings indicate a positive association between childhood neglect, internalizing behaviors, and the adoption of self-blame as CERS. Moreover, the use of self-blame in response to everyday stress partially mediated the relationship between neglect and internalizing behaviors (effect size: .28). Findings support the hypothesis that even in a low risk sample, neglect is associated with internalizing symptoms, and highlight the importance of assessing individual differences in the experience of neglect. Moreover, the mediation effect of the CERSs of self-blame might serve as a potential target for psychotherapeutic interventions aimed at reducing internalizing symptoms.

Child neglect is commonly understood as the failure of caregivers (even without intent) to meet their child’s basic physical, emotional, medical, or educational needs (Gilbert et al. 2009). It can include minor isolated incidents as well as more severe or consistent patterns of failure. Experiences of childhood neglect can therefore be seen as existing on a continuum (Dubowitz 2013) and can have diverse harmful effects on a child’s outcomes (Fonagy et al. 2015) enduring into adulthood (Anda et al. 2006). Child neglect often co-occurs with child abuse (Kim et al. 2017), which involves physical or persistent emotional harm and can have severe adverse effects on the child’s development (Krug et al. 2002).

While extensive research has focused on the impairments associated with childhood neglect in comparison with control groups, there remains a need for studies that examine these associations in community individuals reporting incidents of neglect, some of which are below the “clinical cut-off” but which might nevertheless have negative sequelae. In the present study, we focus on individual differences in response to childhood experiences of neglect in low-risk adolescents, and examine how and whether these experiences relate to internalizing behaviors and the use of different cognitive emotion regulation strategies (CERSs).

## Neglect and Internalizing Symptoms

Neglect is a major risk factor for the emergence of various forms of psychopathology (Egeland et al. 1983; Hecker et al. 2018; Norman et al. 2012; Sturge-Apple et al. 2006; Widom et al. 2007), enduring into adulthood. Prospective and retrospective research has linked neglect with the emergence of internalizing symptoms such as depression or emotional withdrawal (Bolger and Patterson 2001; Hecker et al. 2018; Kim and Cicchetti 2010; Manly et al. 2001; Spratt et al. 2012). For example, it has been shown that emotional neglect in early childhood is positively associated with social withdrawal in middle childhood and negatively with adolescents’ social competence (Shaffer et al. 2009).

The lack of attuned emotional or physical responses from the caregiver, in the context of childhood neglect, may limit or disrupt the child’s emotional and cognitive development (Cook et al. 2005; Dvir et al. 2014; Gould et al. 2012; Hildyard and Wolfe 2002, 2007; Kendall et al. 1998; Maguire et al. 2015; Mills et al. 2011). For example, a comparison between children with no history of neglect, children with a history of neglect, and children with a history of institutional rearing (i.e., having had the experience of early neglect) who were adopted into families of high socioeconomic status showed that the two groups of children with a history of neglect reported significantly more internalizing symptoms than the control group. In the adopted group, there was a positive correlation between internalizing behaviors and the duration of their stay in the neglectful environment. Moreover, both neglect groups showed lower cognitive and language scores compared with nonneglected controls, highlighting the effect of early neglect on cognitive and verbal development (Spratt et al. 2012).

Children who have suffered from neglect, as compared with abuse, have been found to have more limited social relations and higher rates of cognitive deficit and internalizing symptoms, suggesting that, above and beyond the effect of abuse, neglect significantly impacts on individuals’ developmental trajectory (see review by Hildyard and Wolfe 2002; Pollak et al. 2000). In addition, when comparing children who have experienced neglect with children who have experienced both abuse and neglect, the neglect-only group scored significantly lower on vocabulary tests (O’Hara et al. 2015). This reduced verbal performance score suggests a specific pathological outcome relating to neglect: a deficit or absence of sustained social interactions that may have a developmental effect on language skills (O’Hara et al. 2015). In addition, brain imaging analysis has shown a different neural response to threat reactivity, with more distributed cortical reactivity in individuals who have suffered from neglect as compared to a more localized activation among individuals who have experienced abuse (Puetz et al. 2019). However, recent findings also highlight the particularly deleterious effect of emotional abuse on mental health (Cecil et al. 2017).

Despite the consistently powerful findings on the negative effects of neglect, there is still a need for studies that explore its effects on a continuum. Studies that explore individual differences in relation to maltreatment using the childhood trauma questionnaire (CTQ) as a continuous score, have reported a positive association between internalizing symptoms (e.g., Herringa et al. 2013; Huh et al. 2017), emotion dysregulation and psychopathology (Jennissen et al. 2016; Peh et al. 2017). In addition, a study examining the effect of emotional abuse and neglect on a continuum has found a positive relation with internalizing symptoms (Hamilton et al. 2013) and another exploring the continuous effect of the combined score of emotional abuse and neglect found that self-criticism mediated the link between emotional abuse and neglect and romantic satisfaction (Lassri et al. 2016).

Focusing on neglect indices, especially in adolescence, may further our understanding of the underlying mechanisms linking it to the development of psychopathology in later life. As will be discussed further below, adolescence is an especially significant period for the emergence of the negative sequelae of neglect given its importance as a period of biological, cognitive, and psychosocial development (Blakemore 2012).

## Adolescence, Cognitive Emotion Stress Regulation and Internalizing Behaviors

Adolescence is a time of change (Fuhrmann et al. 2015). These significant changes take place in almost every arena (Steinberg and Morris 2001), and the experiences that accompany them generate an emotional, cognitive, and interpersonal stress load that confers vulnerability to mental health problems. During adolescence, a shift in social focus towards peer relations takes place: adolescents experience what their peers think of them extremely powerfully, and are hypersensitive to social rejection, elevating the risk of suffering from internalizing disorders such as social withdrawal and depression (Blakemore 2012). These social changes, taking place alongside neurobiological development, appear to create a particular window of vulnerability for the emergence of mental health disorder. Indeed, most psychopathologies (e.g., depression, anxiety, psychosis, substance use disorders, and eating disorders) first manifest during adolescence (Kessler et al. 2005; Paus et al. 2008).

Critically, biopsychosocial changes during adolescence create heightened demands in relation to self-monitoring and self-regulation capacities, making the ability to regulate stress a potential risk or resilience mechanism in this developmental period (Blakemore and Mills 2014; Garnefski et al. 2002). Adolescence further represents a period of growth in cognitive abilities (Aldwin 2007) and self-regulation, notably through the use and improvement of coping skills that sustain the management of emotion activation and stress (Garnefski et al. 2001). Individuals develop different cognitive approaches, or strategies, to regulate emotions in response to stress: the establishment of such strategies typically settles through the opportunities afforded by adolescent biopsychosocial development (Garnefski et al. 2001).

Empirical studies on cognitive coping have put forward a model of four maladaptive strategies – self-blame, other-blame, rumination, and catastrophizing – and five adaptive strategies – putting into perspective, positive refocusing, positive reappraisal, acceptance, and planning – that adolescents appear to use in response to stressful situations (Garnefski et al. 2005). The maladaptive or adaptive nature of the strategies adopted by an individual can have a long-term impact. For example, the use of strategies such as catastrophizing, self- and other-blame may, in the short term, help regulate distress to a level that is familiar and tolerable, but the entrenched use of this strategy will lead to biased self-appraisals and reduced social functioning psychopathology (Rudrauf and Debbané 2018).

Empirical work on cognitive coping has evidenced the association between maladaptive cognitive emotion regulation strategies (CERSs) and internalizing problems in adolescents (Garnefski et al. 2001, 2002, 2005). For example, individuals who displayed internalizing behaviors reported greater self-blame than individuals with externalizing behaviors (Garnefski et al. 2005). Furthermore, rumination and catastrophizing appear to moderate the link between bullying and anxiety symptoms in adolescence (Garnefski and Kraaij 2014). Research on adult patients experiencing clinical anxiety/depressive symptoms shows that maladaptive CERSs partially mediate the link between childhood maltreatment and the severity of symptoms (Huh et al. 2017).

In addition, studies have consistently found that the relationship between childhood maltreatment and the development of psychopathology symptoms is mediated by difficulties in emotion regulation (Alink et al. 2009; Langevin et al. 2015). For example, emotion dysregulation has been shown to mediate the relationship between adverse childhood experiences, including neglect, and psychopathology (Jennissen et al. 2016), as well as interpersonal difficulties in adulthood (Poole et al. 2018).

From a developmental perspective, the adoption of adaptive emotion regulation strategies is supported through secure interactions with significant others (Eisenberg et al. 2010). Through interactions with responsive caregivers as a source of comfort, infants and children develop their own abilities to self-regulate. Childhood maltreatment in all its forms disrupts the typical development of these skills, either by causing the employment of maladaptive psychological strategies or through adverse effects on neurobiological development (or both) (Dvir et al. 2014). For example, children who have experienced significant physical neglect demonstrate impaired emotion recognition, use fewer adaptive regulation skills, and show more attempts to suppress their emotions compared with control groups (Pollak et al. 2000; Shipman et al. 2005).

Taken together, the developmental framework of emotion regulation and its significance in adolescence highlights the importance of understanding the impact of child maltreatment, and specifically child neglect, on mental health problems in adolescence through the prism of CERSs. However, to our knowledge, there is limited research examining these relationships in low-risk adolescents who have lived through a range of neglectful experiences, from fairly minor to significant in severity. Such an approach may be increasingly important in the context of the ongoing shift in psychological research away from categorical diagnoses toward conceptualizing disorder(s) on continuous or spectral dimensions (Krueger et al. 2018).

## The Present Study

The present study examines the relationship between childhood neglect, CERSs, and internalizing symptoms in a sample of low-risk adolescents. The study attempts to circumvent three issues: first, despite the frequent co-occurrence of multiple types of maltreatment (Kim et al. 2017), most extant studies of neglect do not control for the presence of abuse. Second, despite findings on the effect of neglect on cognitive abilities (O’Hara et al. 2015), to our knowledge, no study to date has focused on CERSs while controlling for cognitive abilities such as verbal functioning. Third, most studies have undertaken group comparisons between maltreated and nonmaltreated individuals, leaving intact the opportunity to assess individual differences across the entire continuum, without being confounded by factors related to clinical populations (e.g., effects of medication, social rupture and isolation linked to psychopathology, comorbidity, etc.).

We hypothesized that: (1) similar to clinical groups, adolescents’ experiences of childhood neglect even below the clinical “cut-off” would be associated with higher reported levels of internalizing problems; (2) replicating previous findings, internalizing problems would be positively correlated with maladaptive CERSs, and negatively with adaptive ones; (3) based on the reported previous mediation effect of CERSs in adults (Huh et al. 2017), CERSs would mediate the link between neglect and internalizing behavior.

## Methods

### Participants and Procedure

A total of 132 community adolescents were recruited through written advertisements and by word of mouth in local schools and youth community centers in Geneva, Switzerland. Inclusion criteria were age between 12 and 19 years, attendance at an age-appropriate school, and no past or current psychiatric treatment and/or neurological conditions, as assessed by a self-report questionnaire. Information concerning the socio-economic status of the participants’ parents was available for 129 adolescents: 6.8% were low socio-economic status, 20.9% were medium–low, 16.3% medium, 17.8% medium–high, 38% high socio-economic status. Information about ethnic origin was available for 105 adolescents: 39% were Swiss, 8.6% French, 7.6% Southern European (Portugal, Spain, Italy), 4.8% Eastern European and Balkan (Russia, Moldavia, Kosovo), 3.8% South American (Brazil, Colombia), 3.15% African (Ghana, Guinea, Ethiopia, Eritrea), 33.3% were of mixed ethnic origin (such as 54.3% European mixed, 22.8% European-South America, 11.4% European-African, 2.8% European-Middle East, 8.5% European-South Asia). Participants received financial compensation, and written consent was obtained from participants or their parents (if under 18 years of age), following a protocol approved by the university ethics committee. Each participant was individually tested in the university lab.

As we were interested in studying typical development, exclusion criteria included verbal and cognitive performance corresponding to IQ <70 (*n* = 9) in the vocabulary and block design subtests of the Wechsler Intelligence Scale (Wechsler Intelligence Scale for Children – Fourth edition; Wechsler 2003 and for participants over the age of 18, the Wechsler Adult Intelligence Scale –Third edition; Wechsler 1997). The final sample consisted of 123 adolescents. The descriptive statistics for the different variables included in our analysis are presented in Table 1.

**Table 1:** Participants’ characteristics

Measure	*M* (*SD*) (*N* = 123)	Range
Females/Males	64/59	–
Age in years	15.88 (1.86)	12.01–18.99
Vocabulary subset	11.48 (2.99)	1–19
Internalizing T-score	52.84 (10.53)	32–82
Internalizing subscales T-score		
Anxiety/Depression	56.48 (7.85)	50–94
Withdrawal	55.30 (7.55)	50–91
Somatic complaints	55.51 (6.96)	50–79
CTQ subscales		
Neglect sum score	17.29 (5.64)	10–35
Abuse sum score	18.38 (5.10)	15–43
CERQ subscales		
Self-blame	9.54 (3.14)	4–19
Other-blame	7.80 (2.81)	4–20
Rumination	11.55 (3.55)	5–20
Catastrophizing	8.42 (3.59)	4–19
Acceptance	13.70 (3.44)	6–20
Positive refocusing	11.52 (3.97)	4–20
Positive reappraisal	13.47 (3.73)	4–20
Putting into perspective	13.72 (3.90)	5–20
Refocus on planning	13.95 (3.51)	5–20

*CERQ* Cognitive emotion regulation questionnaire, *CTQ* Childhood trauma questionnaire

### Instruments

#### Behavioral and Emotional Problems

To evaluate participants’ psychopathology, we used the Youth Self-Report (YSR; for individuals aged 11–17; Achenbach 1991) and its adult equivalent, the Adult Self Report (ASR; for individuals above 17 years; Achenbach and Rescorla 2003) questionnaire. Both are designed to assess symptoms in the past 6 months using a 3-point scale (0 = *not true* to 2 = *very true*). The YSR and the ASR were validated in French. We focused on the internalizing scale reflecting three subscales, which evaluate the presence of the following difficulties: Anxious/Depressed, Withdrawn/Depressed and Somatic Complaints (Cronbach’s α: Internalizing ASR = .88; YSR = .82). For each subscale, a total T-score is computed. A T-score of 65 or 70 in the specific subscales represents the cut-off for clinically significant symptomatology. Of the total sample, 12% fell into the clinical range of internalizing symptoms (T score > 64), 3% fell into the clinical range of withdrawal symptoms or somatic complaints and 5% into the clinical range for anxiety and depression (T scores >70).

#### Childhood Neglect and Abuse

To evaluate individuals’ experience of childhood trauma, we used the French version of the Childhood Trauma Questionnaire (CTQ; Bernstein et al. 1994; Paquette et al. 2004), a 28-item self-report questionnaire validated for use in clinical and nonclinical populations. Individuals are asked to indicate on a 5-point Likert scale how often (1 = *never*, 5 = *frequently*) they experienced emotional, physical, and sexual abuse, and emotional or physical neglect in childhood. The French version has been validated with French-speaking populations (Paquette et al. 2004). In this study, the emotional and physical neglect subscales were summed to provide a total score of neglect and the emotional, physical, and sexual abuse subscales were summed to give a total score of abuse (Cronbach’s α = .70 and .72, respectively). Within our sample, 16% reported moderate to severe emotional neglect (scale score > 15), 9% moderate to severe physical neglect (scale score > 10), 5% moderate to severe physical abuse (scale score > 10), 4% moderate to severe emotional abuse (scale score > 13), and 3% moderate to severe sexual abuse (scale score > 8). These percentages are lower than those reported in international and European surveys (Moody et al. 2018).

#### Cognitive Emotion Regulation Questionnaire

To evaluate the cognitive emotion regulation strategies (CERSs) used in response to a negative or traumatic event (Garnefski et al. 2001), we used the French version of the Cognitive Emotion Regulation Questionnaire (CERQ; Jermann et al. 2006). This version was validated in a community sample of adolescents and is comparable with the original English questionnaire (d’Acremont and Van der Linden 2007). The CERQ is a 36-item questionnaire that uses a 5-point Likert scale to assess nine strategy subscales: self-blame, rumination, catastrophizing, blaming others, acceptance, positive refocusing, refocus on planning, positive reappraisal, and putting into perspective (Cronbach’s α = .74, .64, .72, .83, .66, .80, .75, .80, and .78, respectively). Subscale scores can range from 4 to 20, with higher subscale scores indicating greater frequency of use of the specific cognitive strategy. Comparing the present sample scores on the CERQ sub-scales with those reported in d’Acremont and Van der Linden (2007), revealed that all fell within the average score.

#### Verbal Cognitive Functioning

To control for cognitive verbal functioning, we used the French version of the vocabulary subtest of the Wechsler Intelligence Scale for Children – Fourth edition (WISC; Wechsler 2003). For participants over the age of 18, the Wechsler Adult Intelligence Scale – Third edition (WAIS-III; Wechsler 1997) was used. The vocabulary subtest measures word knowledge, language development, and concept understanding.

### Data Analysis

We first ran descriptive statistics and as the variables of interest (i.e., neglect, internalizing behaviors and CERQ subscales) violated normality, we used nonparametric tests. First, we used the nonparametric Mann–Whitney U test to compare male and female participants on variables of interest (internalizing behaviors, experience of neglect and abuse, CERSs). Second, we ran a partial Spearman correlation analysis using R, conducted between neglect and internalizing behaviors, controlling for age, sex, abuse, and Wechsler’s WISC/WAIS vocabulary subtest score. Third, Spearman’s rho correlation analyses were performed to assess the associations between neglect, CERQ results, and internalizing behaviors. Results were corrected for multiple comparisons using a 5% false discovery rate (FDR), based on the sequential Benjamini–Hochberg FDR correction algorithm (Benjamini and Hochberg 1995). Finally, based on the correlation analysis, and in order to test the mediation hypothesis, we used SPSS version 25 combined with Hayes’s PROCESS macro (Hayes 2018). This macro uses bootstrapping (10,000 resamples) for testing mediation effects and is considered one of the most valid and powerful simulation methods (Hayes 2009).

## Results

### Descriptive and Correlation Analysis

The nonparametric Mann–Whitney U test for two independent groups revealed that males and females did not significantly differ in most of the variables [(i.e., internalizing behaviors (*p* = .79), neglect (*p* = .10) and abuse experience (*p* = .46), or the use of most of the CERSs (*ps* > .36) except the use of rumination as a strategy: our findings suggested that females used this strategy more than males (male *M* = 10.56, *SD* = 3.25; female *M* = 12.43, *SD* = 3.22, *p* = .001)]. Partial correlation analysis between neglect and internalizing behaviors, controlling for abuse, sex, age, and verbal cognitive functioning, yielded a positive correlation (*r* = .27, *p* = .002). In addition, when examining the Spearman correlation coefficients (Table 2; FDR corrected) between neglect, internalizing behaviors and CERSs, there were positive correlations between neglect and self-blame (*r* = .30, *p* = .003, 95% CI [0.13, 0.45]), and neglect and catastrophizing (*r* = .25, *p* = 0.02, 95% CI [0.07, 0.40]). There were no significant correlations between neglect and any of the adaptive CERSs. There were also positive correlations between internalizing behaviors and the CERSs of self-blame (*r* = .38, *p* = .00002, 95% CI [0.21, 0.52]), catastrophizing (*r* = .22, *p* = .01, 95% CI [0.04, 0.38]), rumination (*r* = .26, *p* = .003, 95% CI[0.08, 0.42]), and a positive significant trend with the tendency to blame others (*r* = .20, *p* = .07, 95% CI [0.02, 0.30]). There were no significant correlations between internalizing and any of the adaptive CERSs.

**Table 2:** Summary of Spearman intercorrelations of neglect, internalizing behaviors and CERQs (*N* = 123, FDR corrected)

	1	2	3	4	5	6	7	8	9	10	11
1. Internalizing behavior	–	.40^**^	.38^**^	.20	.26^*^	.22^*^	.10	.00	.03	.04	.03
2. Neglect		–	.30^**^	.13	.18	.25^*^	.15	.07	.04	.04	.05
3. CERQ – self-blame			–	.38^**^	.45^**^	.35^**^	.23^*^	.00	.17	.17	.20^*^
4. CERQ – other-blame				–	.22^*^	.37^**^	.03	.16	.11	.06	.14
5. CERQ–rumination					–	.44^**^	.38^**^	.26^**^	.35^*^	.32^**^	.30^**^
6. CERQ – catastrophizing						–	.09	.18	.05	−.01	.00
7. CERQ – acceptance							–	.40^**^	.45^**^	.53^**^	.27^*^
8. CERQ – positive refocusing								–	.49^**^	.41^**^	.32^**^
9. CERQ – positive reappraisal									–	.61^**^	.44^**^
10. CERQ – putting into perspective										–	.20
11. CERQ – refocus on planning											–

*CERQ* Cognitive emotion regulation questionnaire, *FDR* False discovery rate

*p < 0.05

**p < 0.001

### Mediation

**Fig. 1:**
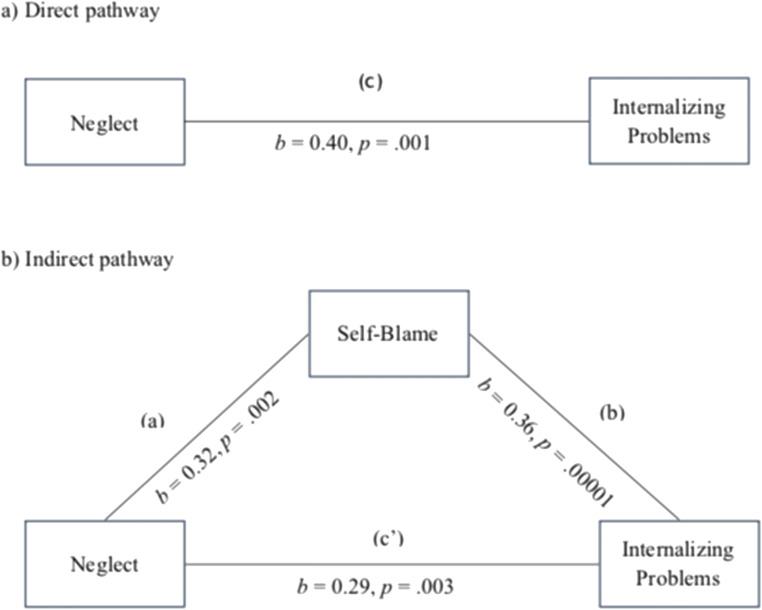
Mediation model. With neglect as independent variable, self-blame as mediator, and internalizing problems as dependent variable controlling for age, sex, abuse and Wechsler’s WISC/WAIS-III vocabulary subtest score

Based on the correlation results, we tested whether the CERS self-blame score mediated the effect of neglect on internalizing symptoms. Results indicated that when controlling for age, sex, abuse, and verbal cognitive score, neglect was a significant predictor of self-blame (path a in Fig. 1; *b* = .32, *SE* = .10, *p* = .002, 95% CI [0.12, 0.52]), and self-blame was a significant predictor of internalizing behaviors (path b in Fig. 1; *b* = 0.36, *SE* = .09, *p* = .00001, 95% CI [0.19, 0.53]). The total effect of neglect on internalizing behaviors (*b* = .40, *SE* = .10, *p* = .001, 95% CI [0.21, 0.61]) was reduced by about 11% when self-blame was accounted for (path c’ in Fig. 1; *b* = .29, *SE* = .09, *p* = .003, 95% CI [0.09, 0.48]), indicating partial mediation. The indirect coefficients were significant (*b* = .12, 95% CI [0.03, 0.23]) and the mediation effect size, calculated as the ratio between the indirect and the total effect (Wen and Fan 2015), was found to be .28.

Using the same method, we also examined whether the CERS of catastrophizing mediated the effect of neglect on internalizing behaviors, as the use of this strategy was also positively correlated with both neglect and internalizing behaviors. However, indirect coefficients were not significant (*b* = .04, 95% CI [−0.005, 0.10]), indicating no mediation.

### Post-hoc Analysis

#### Subscales of the Internalizing Dimension

To further examine the mediation effect of self-blame on the relationship between neglect and internalizing behaviors, we conducted similar mediation analysis using the subscales of the internalizing dimension (i.e., withdrawal, depression/anxiety, and somatic complaints). This analysis yielded the finding that self-blame fully mediated the link between neglect and withdrawal and partially mediated the link with depression/anxiety (Table 3). However, there was no effect in relation to somatic complaints.

**Table 3:** Post-Hoc tests of a. indirect effects in the model of neglect and internalizing dimension, mediated by self-blame, and b. subtypes of neglect and internalizing behaviours. In both models age, sex, abuse, and Wechsler’s WISC/WAIS-IV vocabulary subtest score were controlled

Path	b	*SE*	95% CI	
			Lower	Upper
a.				
Neglect → SB → Withdrawal	.11	.06	0.03	0.25
Neglect → SB → Anxiety/Depression	.14	.06	0.02	0.28
Neglect → SB → Somatic complaints	.06	.04	−0.07	0.15
b.				
EN → SB → Internalizing behaviours	0.08	0.04	0.001	0.19
PN → SB → Internalizing behaviours	0.12	0.05	0.04	0.23

*CI* Confidence intervals; *EN* Emotional neglect; *PN* Physical neglect, *SB* Self-blame

#### Subtypes of Neglect

To further examine the mediation effect of self-blame on the relationship between subtypes of neglect and internalizing behaviors, we conducted a similar mediation analysis using the subtypes of neglect before aggregating them to a total score (i.e., emotional and physical neglect). This analysis yielded the finding that self-blame fully mediated the link between physical neglect and internalizing, and partially mediated the link with emotional neglect (Table 3).

## Discussion

In the present study, we examined in a cross-sectional design the relationships between childhood neglect, CERSs, and internalizing symptoms in low-risk adolescents. We observed that the association between childhood neglect and internalizing behaviors is specifically mediated by the CERS self-blame. Our findings support our first hypothesis that, similarly to clinical samples (Bolger and Patterson 2001; Hildyard and Wolfe 2002; Kim and Cicchetti 2010; Manly et al. 2001; Spratt et al. 2012), low-risk adolescents’ reports of perceived childhood neglect experiences are associated with current internalizing symptoms. Given that experiences of childhood abuse were controlled for in this study, this finding highlights the specific effect of neglect.

The second hypothesis, concerning the relationship between internalizing symptoms and CERSs, was partially supported. Findings showed a positive relationship between internalizing symptoms and all maladaptive CERSs (e.g., rumination, catastrophizing, self-blame, and other-blame) but not with adaptive ones. This finding is inconsistent with previous reports of negative associations between depression severity and adaptive CERSs in adults with depressive disorders (Huh et al. 2017). However, it is consistent with previous studies reporting that adolescents with internalizing symptoms score higher on maladaptive CERSs compared with a control group, but do not differ in adaptive ones (Garnefski et al. 2005). While it is necessary to explore further the null relation between internalizing symptoms and adaptive CERSs, the pronounced recourse to maladaptive strategies to regulate stress might suggests that psychological interventions targeting emotion-regulation skills may be indicated for vulnerable adolescents (Loevaas et al. 2019; Rossouw and Fonagy 2012; Sharp et al. 2009; Sukhodolsky et al. 2004; Young et al. 2019).

In addition, only the CERSs related to self-blame and catastrophizing were positively associated with both neglect and internalizing symptoms, allowing us to test the mediation hypothesis. Interestingly, the association between neglect and internalizing behavior is mediated by the CERS of self-blame and not by catastrophizing. This is consistent with previous findings that self-blame partially mediates the effect of childhood neglect on nonsuicidal self-injury in adulthood (Swannell et al. 2012), as well as the link between childhood traumatic experiences and symptom severity in adults with depression (Huh et al. 2017). However, it further suggests that this relationship is present at a normative level and not uniquely present in the context of clinical symptoms. This finding may be attributable to the particular nature of childhood neglect, which implies a lack of emotional or physical care by attachment figures (Leeb et al. 2011; Mennen et al. 2010). The child in a neglectful environment experiences reduced exposure to the caregiver responses and interactions; as a consequence, the child may be left with little option other than to adopt self-blame as there is no “other” to take the blame or to be blamed for these experiences.

Further post-hoc analysis within the internalizing dimension found that self-blame fully mediates the link between neglect and the withdrawn subscale, and partially mediates the relation between neglect and depression/anxiety, but not somatic complaints. These specific paths are intriguing and consistent with a different but related conceptualization of self-criticism as a stance augmented in neglectful familial environments (Shahar 2015): the experience of neglect might lead to the use of strategies that are directed inward (e.g., self-blame) at moments of heightened stress, although this path would require longitudinal investigations. The increased impact and value of social relations in adolescence (Blakemore 2008) may heighten the intensity of the affect associated with self-blame, generating greater distress and potentially leading to increased social impairment.

Contrary to findings that adaptive CERSs may mediate the link between neglect and adult anxiety/depression in a group of clinical adults (Huh et al. 2017), we did not find any association between neglect and adaptive CERS dimensions (e.g., positive refocusing or reappraisal). The difference could be accounted for by our use of a general population sample rather than a clinical one, as well as the difference in age in the samples studied, and should be further investigated in future research focusing on adaptive resilience mechanisms using longitudinal designs.

From a developmental perspective, the absence of attuned emotional responses in the context of childhood neglect in early relationships may limit the child’s capacity to regulate emotions and to develop adaptive mental representations of themselves and others, augmenting the risk for psychopathology (Cook et al. 2005; Feldman and Greenbaum 1997; Fonagy et al. 2017; Fonagy and Target 2002; Toth et al. 1997). Put differently, in terms of cognitive behavioral learning theory (Bandura 1986), such limited or maladaptive regulation strategies may also affect the individual’s ability to regulate stress during adolescence. Influenced by past experiences of neglect, the individual may have learned to blame him/herself for the lack of social support, leading to the adoption of more general self-blame strategies that might prevent him/her from integrating or appraising new information. This can in turn lead to withdrawal, anxiety and/or depression (Debbané et al. 2016; Fonagy et al. 2017; Rudrauf and Debbané 2018). Further, such strategies may also generate a vicious cycle in which individuals with internalizing symptoms are likely to continue to process information in a dysfunctional manner; this biased acquisition and processing style can contribute to the maintenance of psychopathology (Beck 2008) and limit the capacity to use adaptive strategies.

From a theoretical developmental perspective, using Erikson’s psychosocial stages (Erikson 1956) it might be that experiences of neglect may reduce feelings of trust and create higher levels of guilt and shame. These negative resolutions of the psychosocial crises are thought to reawaken with particular acuteness during adolescence, compromising the work of adaptively resolving their “Ego identity” and leading to lower levels of autonomy and more isolation, which might express itself in internalizing symptoms.

Certain limitations in the present investigation should be acknowledged. First, the modest size of our sample should be noted. We hope that future studies will replicate these findings using a larger group. In relation to this limitation, it should also be observed that our sample is a convenience sample and we cannot rule out selection bias, which might limit the generalizability of the findings. Second, as this study uses a cross-sectional design, and although the direction of the mediation is based on the assumption that childhood experience refers to the past, while CERS and internalizing behaviors are current experiences – with the first being more state-related and the later more symptom-related – we cannot rule out different directions. For example, it might also be that a higher level of internalizing would lead to extensive use of self-blame strategies, or recurrent memories of neglect. Future longitudinal studies should test these directions and investigate the long-term effects of neglect or CERSs on internalizing behavior. Third, as neglect was assessed using a retrospective self-report tool, reporting bias cannot be ruled out. Fourth we did not assess other forms of emotion dysregulation (e.g., general emotion regulation; Gross 1998) or attachment styles that could have accounted for the present effects. Likewise, our findings focused only on the adolescents’ self-report. Using stress-induced experimental designs or other related biophysiological variables, such as implicit emotion regulation (Etkin et al. 2011), might shed light on possible moderation effects that could have further implications in terms of resilience or psychotherapeutic interventions. Finally, it should be noted that in an attempt to explore neglectful experiences on a continuum, stressing the importance of examining below the radar neglectful experiences such as feelings of being unloved by a caregiver, may create a risk of overpathologizing, and researchers as well as clinicians should take this into consideration.

To conclude, to the best of our knowledge, this investigation is the first to demonstrate the association between experiences of neglect that are below the clinical cut-off, coping mechanisms in relation to stress in adolescence, and the emergence of psychopathology, in particular internalizing symptoms. Moreover, our finding on the relationship between the CERS of self-blame and internalizing behavior in nonclinical adolescents may point to a potential risk factor that could inform psychological interventions.
